# Sex, age, deprivation and patterns in life expectancy in Quebec, Canada: a population-based study

**DOI:** 10.1186/1471-2458-10-161

**Published:** 2010-03-25

**Authors:** Nathalie Auger, Carolyne Alix, Geng Zang, Mark Daniel

**Affiliations:** 1 Études et analyses de l'état de santé de la population, Institut national de santé publique du Québec, Montréal, Québec, Canada; 2 Research Centre of the University of Montreal Hospital Centre, Montréal, Québec, Canada; 3 Department of Social and Preventive Medicine, University of Montréal, Montréal, Québec, Canada; 4 School of Health Sciences, University of South Australia, Adelaide, Australia

## Abstract

**Background:**

Little research has evaluated disparities in life expectancy according to material deprivation taking into account differences across the lifespan between men and women. This study investigated age- and sex-specific life expectancy differentials related to area-level material deprivation for the province of Québec, Canada from 1989-2004.

**Methods:**

Age- and sex-specific life expectancy across the lifespan was calculated for three periods (1989-1992, 1995-1998, and 2001-2004) for the entire Québec population residing in 162 community groupings ranked according to decile of material deprivation. Absolute and relative measures were calculated to summarize differences between the most and least deprived deciles.

**Results:**

Life expectancy differentials between the most and least deprived deciles were greatest for men. Over time, male differentials increased for age 20 or more, with little change occurring at younger ages. For women, differentials increased across the lifespan and were comparable to men at advanced ages. Despite gains in life expectancy among men relative to women, differentials between men and women were greater for most deprived relative to least deprived deciles.

**Conclusions:**

Similar to the US, differentials in life expectancy associated with area-level material deprivation increased steadily in Québec from 1989-2004 for males and females of all ages. Differentials were comparable between men and women at advanced ages. Previous research indicating that life expectancy differentials between most and least deprived areas are greater in men may be due to a focus on younger age groups.

## Background

Research examining area-based, or geographic, trends in mortality has grown over the past decade [1-15]. Studies have considered mortality outcomes [2-5,9,10,13-15] and life expectancy [1,6-8,11,12,14,15]. Life expectancy is an easily understood measure of population mortality [7,12,16] for which summary measures of disparities between most and least deprived areas can be calculated [11]. Such information can contribute importantly to understanding the nature of changes in life expectancy over time [17].

Studies indicate that life expectancy differentials between the most and least materially deprived areas of the US, UK and New Zealand have recently increased, and may be continuing to do so [1,6,11,14]. Whether similar patterns are present in Canada is unclear [15], although some provinces have reported widening gaps in premature mortality [4,5]. An evaluation of Canadian life expectancy differentials could shed light on this research gap, and would be especially meaningful because of Canada's relationship to the US. Canada shares close economic and cultural ties with the US, but differs significantly in maintaining a strong social welfare system, including universal health insurance.

A related issue is whether patterns in life expectancy disparities associated with material deprivation have changed for age groups across the lifespan. Most studies tend to focus on differentials in life expectancy at birth, or on premature mortality, thus excluding older age groups. Differentials in life expectancy at older ages have rarely been evaluated, despite individual-level research indicating that the rising differentials might be substantially driven by population growth among individuals aged 65 and above [18,19]. The only area-based study to examine a spectrum of ages found that life expectancy differentials increased over time between the least and most deprived areas of the US for all ages in both sexes, yet were smallest for the population 70 years and over [11]. Age-specific patterns for life expectancy disparities are not known for other countries.

Sex-based patterning of area-based life expectancy differentials is an important related question that little research has explicitly evaluated. Individual-level research suggests that the lower socio-economic differentials frequently observed in women may be biased, primarily because of a focus on mortality in young individuals (an age group in which male differentials predominate) [18]. In other words, socio-economic differentials might be greater among women at older ages. However, life expectancy differentials between the most and least deprived areas of the US at older ages are still greater among men than women [11].

Another way to compare women and men is to look at the difference in life expectancy between both sexes. In England, differences in life expectancy at birth are greater in more deprived compared to less deprived areas, and have also increased over time [7]. Trends in female-male differentials associated with area-based deprivation remain to be studied for other countries.

Given the gaps in the literature, this study aimed to advance knowledge of how material disadvantage influences life expectancy by examining sex- and age-specific differentials related to area-based deprivation in Québec, a large Canadian province.

## Methods

### Data and life expectancy estimates

Deaths were extracted from the Québec health ministry vital statistics files. Age-specific mortality rates were calculated according to sex for three 4-year periods (1989-1992, 1995-1998 and 2001-2004). Deaths before 1989 were not examined because areas with stable borders and population estimates were not available. The 2-year span between periods was not analysed to increase comparability with US analyses [11] and to avoid diluting the difference between adjacent periods (we verified that analyses using all years did not alter results). Population counts for the middle year of each study period were obtained from census projections, and were used to calculate mortality rates. Population counts were adjusted for under-enumeration [20]. Life expectancy was estimated using the standard life table method by calculating the age-specific mortality of twenty age groups (<1, 1-4, 5-9, ., 85-89, 90+ years) and converting to life table probabilities of dying [21]. The probability of dying in the first year of life was estimated from the infant mortality rate, and a probability of 1 was used for the last age group [22].

### Classification of areas

Local community services centres (CLSC, N = 166) recorded in the Québec death file were used as they represent meaningful areas for which disadvantage varies. CLSCs with borders fixed through time are available, making them suitable for use in area-based analyses [11]. CLSC population statistics are also available. The 1996 population size of CLSCs (mean 42,212 inhabitants; range 1,355-133,475) is comparable to US counties [11], facilitating comparisons between Québec and the US.

A composite index of material deprivation was used to grade CLSCs. A deprivation index for 1996 Census enumeration areas (N = 9058) is available as quintiles [23] and has been widely used in provincial studies [5,24-28]. The index is derived by categorizing as quintiles the output from a principal component analysis of census information on persons without a high school diploma, employment, and average income (which distributes enumeration areas according to a normal distribution). This methodology is similar in principle to the Townsend index [29]. We used the deprivation index of enumeration areas to calculate a population-weighted index for each CLSC. Four northern CLSCs with incomplete or missing census data were excluded because deprivation indices could not be calculated. The remaining CLSCs (N = 162) were ranked as population-weighted deprivation deciles. The 1996 classification of CLSC deciles (obtained from the 1996 deprivation index) was used for all three study periods under the assumption that the distribution of CLSC deprivation has been stable over time. This approach is supported by studies showing that the broad geographical distribution of deprivation has changed little over the past decades in the US and Britain [8,11,30].

Covariates included CLSC rural/urban status (rural, semi-urban, urban) and presence of Aboriginal reserves (yes, no). These two indicators were calculated using Statistics Canada's 2001 classification for dissemination areas (the 2001 equivalent of 1996 enumeration areas). The categories were generated from the proportion of dissemination areas classified as rural or Aboriginal reserves within CLSCs. A CLSC was considered rural when 100% of dissemination areas were rural, semi-urban when 1 to 99% of dissemination areas were rural, and urban when 0% of dissemination areas were rural [31]. A CLSC was said to contain Aboriginal reserves when at least one of its dissemination areas was located on an Aboriginal reserve. Covariates were selected based on previous research addressing CLSC socio-economic status and health [31,32].

### Statistical Analysis

Period-specific comparisons between deciles representing the most and least materially deprived areas were examined for life expectancy at birth and at 65 years according to sex using the 1) *absolute difference *in life expectancy between bottom and top deciles, 2) *slope index of inequality *[33], 3) *relative index of inequality *[34], and 4) *adjusted slope index of inequality*. The *slope index of inequality *corresponds to the coefficient obtained by regressing mean life expectancy on a transformation of deprivation deciles. The transformed decile values are based on a score for the midpoint of the decile's range in the cumulative distribution of the population, and are treated as a continuous variable in the regression [33]. The *slope index of inequality *is interpreted as the absolute difference in life expectancy between the hypothetical bottom (0^th ^percentile) and top (100^th ^percentile) of the cumulative socioeconomic distribution. The *relative index of inequality *is obtained by dividing the *slope index of inequality *with the mean life expectancy over all deciles [34], and is a measure of the proportionate increase in life expectancy between the hypothetical bottom and top of the cumulative socioeconomic distribution. We computed an *adjusted slope index of inequality *in which life expectancy was regressed on the transformed decile score using individual CLSCs (not deciles) as the unit of analysis, adjusting for CLSC rural/urban and aboriginal classification. The assumptions of linear regression were verified. Nine CLSCs were excluded from the regression because of unstable life expectancy estimates due to small numbers of deaths [35]. We verified that trends were not due to differential population growth by recalculating the *slope index of inequality *for the last study period using the population distribution of the previous study periods [36].

Absolute measures of the change *over time *in life expectancy at birth and 65 years were calculated as the difference in life expectancy between the last and first period for each decile.

To measure female-male gaps in life expectancy at birth and 65 years and changes over time, we calculated the period-specific difference in life expectancy between women and men for each decile, and verified results with the *adjusted slope index of inequality*. We also calculated the absolute difference in the male-female gap between the last and first periods for each decile.

For life expectancy at all ages, we calculated the period-specific absolute difference between the least and most deprived deciles, and illustrated period-to-period differences in an age and sex population pyramid graph. We computed the overall life expectancy for each period (all deciles together), and compared results to the *slope index of inequality*.

Analyses were performed using SPSS version 12.0.1. This study was conducted as part of the Québec population health surveillance activities mandated by the health ministry and approved by the Comité d'éthique de santé publique.

## Results

Individual components of the deprivation index showed relatively large socio-economic differences between the most and least deprived deciles (Table 1). In the most deprived decile, the number of deaths decreased over time among men despite a total population increase, and increased among women despite a stable population size. In the least deprived decile, the number of deaths increased over time (along with the total population) for both sexes. In both sexes, population increases over time were greater for the least deprived relative to most deprived decile.

**Table 1:** Descriptive characteristics of local community service center (CLSC) deciles, Québec, 1989-2004*

	**Least deprived**	**Intermediate**^**†**^	**Most deprived**	**All deciles**
	
**Deprivation index**				
Mean (range)^†^	1.49 (1.04-1.79)	2.81 (2.75-2.87)	4.50 (4.16-5.00)	3.35 (1.04-5.00)
**Total population, male**				
1989-1992	340 329	327 517	376 361	3 464 734
1995-1998	347 685	336 187	381 197	3 575 749
2001-2004	358 817	349 017	381 537	3 682 658
**Deaths. Male**				
1989-1992	9 406	9 327	14 239	107 152
1995-1998	9 636	9 756	14 467	111 919
2001-2004	9 758	9 840	13 979	111 198
**Total population, female**				
1989-1992	364 761	343 411	373 138	3 569 497
1995-1998	374 038	352 649	374 833	3 685 014
2001-2004	385 345	365 553	373 733	3 781 985
**Deaths, female**				
1989-1992	9 130	8 199	10 351	88 041
1995-1998	10 260	9 692	11 625	101 668
2001-2004	11 116	10 271	12 233	109 508
**Infant mortality rate**				
1989-1992	5.2	6.4	7.0	6.1
1995-1998	4.5	4.3	6.1	5.3
2001-2004	4.0	3.8	5.0	4.6
**Population per CLSC**				
Mean	62 430	54 941	19 326	42 212
Range	31 541-107 905	3 645-93 569	2 540-52 099	1 355-133 475
**Unemployment rate**				
Mean	6.3	8.1	14.8	8.4
Range, CLSC	4.5-9.6	5.2-12.9	4.8-34.1	4.5-34.1
**Average household income, $CAN**				
Mean	70 715	48 618	38 314	50 748
Range, CLSC	44 228-102 101	39 771-57 495	28 129-50 385	28 129-102 101
**Percent no high school diploma**				
Mean	17.2	33.7	45.4	31.7
Range, CLSC	8.4-22.2	29.8-38.6	37.1-63.6	8.4-63.6
**Proportion rural CLSCs**				
All rural	0.0	8.3	72.2	30.2
Some rural (i.e., semi-urban)	9.1	8.3	13.9	31.5
No rural (i.e., urban)	90.9	83.3	13.9	30.2
**Proportion aboriginal CLSCs**	0.0	25.0	19.4	17.9

* Only three deciles are shown as patterns in nearby deciles tended to be similar.

† Population weighted mean and range for CLSCs in the decile.

‡ Refers to decile 6 (decile 1 = most deprived, decile 10 = least deprived)

Life expectancy at birth was lowest in the first study period (1989-1992) among males in the most deprived decile (72.3 years), and highest in the last study period (2001-2004) among females in the least deprived decile (84.4 years, Table 2). Life expectancy at 65 years ranged from a low of 14.4 years in the first study period among men in a mid-decile to a high of 22.2 years in the last study period among women in the least deprived decile.

**Table 2:** Life expectancy at birth and at 65 years according to sex and deprivation decile, and female-male difference in life expectancy according to deprivation decile, Québec, 1989-2004

	**Life expectancy at birth**				**Life expectancy at 65 years**			
	**1989-1992**	**1995-1998**	**2001-2004**	difference*	**1989-1992**	**1995-1998**	**2001-2004**	difference*
	
**Females**								
Decile 10 (least deprived)	82.4	83.2	84.4	2.0	20.9	21.2	22.2	1.4
Decile 1 (most deprived)	80.0	80.4	81.4	1.4	19.6	19.5	20.2	0.6
All deciles	80.7	81.0	82.2	1.4	19.8	19.8	20.6	0.8
**Measures of inequality**								
Absolute difference between decile 1 and 10	2.4	2.8	3.0		1.3	1.7	2.0	
Slope index of inequality (p-value)^†^	1.8	1.9	2.1		0.7	0.7	0.9	
	(0.008)	(0.028)	(0.022)		(0.115)	(0.25)	(0.216)	
Relative index of inequality^‡^	0.02	0.02	0.03		0.04	0.04	0.04	
Adjusted slope index of inequality (p-value)^§^	1.8	1.8	2.0		0.5	0.5	0.7	
	(0.013)	(0.002)	(0.012)		(0.413)	(0.218)	(0.485)	
**Males**								
Decile 10 (least deprived)	75.9	77.6	79.8	4.0	16.4	17.0	18.7	2.3
Decile 1 (most deprived)	72.3	73.3	75.6	3.3	14.9	15.4	16.6	1.7
All deciles	73.5	74.7	76.9	3.4	15.1	15.5	16.9	1.8
**Measures of inequality**								
Absolute difference between decile 1 and 10	3.6	4.3	4.3		1.5	1.7	2.1	
Slope index of inequality (p-value)^†^	3.1	3.7	3.7		0.9	1.1	1.4	
	(0.001)	(0.002)	(0.001)		(0.132)	(0.100)	(0.060)	
Relative index of inequality^‡^	0.04	0.05	0.05		0.06	0.07	0.08	
Adjusted slope index of inequality (p-value)^§^	3.3	3.9	3.7		1.0	1.2	1.5	
	(<0.000)	(<0.000)	(<0.000)		(0.226)	(0.055)	(0.005)	
**Difference between females and males**								
Decile 10 (least deprived)	6.5	5.5	4.6	-1.9	4.5	4.2	3.6	-0.9
Decile 1 (most deprived)	7.8	7.1	5.8	-2.0	4.7	4.2	3.6	-1.0
All deciles	7.2	6.3	5.3	-1.9	4.7	4.3	3.7	-1.0
**Measures of inequality**								
Absolute difference between decile 1 and 10	-1.3	-1.5	-1.2		-0.2	0.0	-0.1	
Slope index of inequality (p-value)^†^								
Relative index of inequality^‡^								
Adjusted slope index of inequality (p-value)^§^	-1.6	-2.1	-1.7		-0.5	-0.7	-0.8	
	(0.003)	(0.000)	(0.000)		(0.210)	(0.052)	(0.008)	

* Difference in life expectancy between periods 2001-2004 and 1989-1992 (an absolute measure of change over time). Differences may not add up because of rounding.

† Absolute difference in life expectancy between the bottom and top deciles, obtained from regression of mean life expectancy on mean relative rank of deciles.

‡ Proportionate increase in life expectancy between the highest and lowest deciles.

§Absolute difference in life expectancy between the bottom and top deciles, adjusted for rural-urban classification and presence of Aboriginal areas (refers to the coefficient obtained from linear regression of life expectancy of CLSCs - not of deciles - on the mean relative rank of deciles).

### Period-specific comparisons and patterns over time

Summary measures of inequality between the most and least deprived deciles (*i.e.*, absolute difference, slope index of inequality, relative index of inequality, adjusted slope index of inequality) were more strongly related to life expectancy at birth than at 65 years, for all three study periods (Table 2). These findings were present for both sexes, although disparities were greater among males than females for life expectancy at birth. Summary statistics indicated differentials for life expectancy at birth increased over time in both sexes. These findings were consistent with the decile-specific difference in life expectancy between the last and first study periods; the increase over time was largest for the least deprived decile (males 4.0, females 2.0 years) compared to the most deprived decile (males 3.3, females 1.4 years). Period-specific summary measures of inequality suggested a plateau in the middle study period (1995-1998) among males. Among females, however, period-specific measures of inequality increased steadily over time with less evidence of a plateau.

Patterns for life expectancy at 65 years were less clear. Summary measures of inequality grew from period to period, but were not statistically significant with the exception of men in the last study period, suggesting that differentials in life expectancy at 65 years among men may be emerging. Like life expectancy at birth, there has been a consistent increase in life expectancy at 65 years for each decile between 1989-1992 and 2001-2004, which was largest for the least deprived decile (men 2.3, women 1.4 years) compared to the most deprived decile (men 1.7, women 0.6 years).

### Additional male-female comparisons

Summary measures of absolute inequality showed that the overall difference in life expectancy at birth between top and bottom deciles was twice as large for males than for females in all study periods, indicating that absolute inequality is greater among males (Table 2). However, the difference in life expectancy at birth *between females and males *has decreased steadily over time for each decile (Table 2, bottom third of table), indicating that life expectancy among males is increasing relatively faster than females irrespective of decile. In other words, even though overall life expectancy is higher for women than men, the extent of the rise in life expectancy over time has been greater for men than women in all deprivation deciles. Nonetheless, the adjusted slope index of inequality for the female-male difference between the most and least deprived deciles was statistically significant and negative in all study periods, indicating that the gap between men and women is larger in most deprived than in least deprived areas.

The difference in life expectancy at 65 years between women and men also decreased over time (Table 2, bottom third of table), meaning life expectancy in older men has also been catching-up to women (as there has been an overall increase in life expectancy at 65 years in both sexes). Unlike life expectancy at birth, however, the adjusted slope index of inequality for the female-male difference between the most and least deprived areas was statistically significant in the last period only.

### Patterns in life expectancy across the lifespan

**Figure 1:**
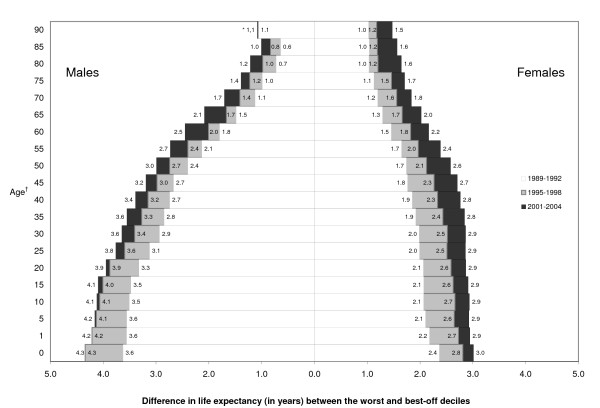
**Trends in the life expectancy gap between most and least deprived areas according to sex and age, Québec, 1989-2004**. * Life expectancy at 90 years among males was the only category for which the difference between the least and most deprived deciles did not increase. In 1995-1998, the difference decreased to 0.7 years but returned in 2001-2004 to the difference observed in 1989-1992 (*i.e*., 1.1 years). † Refers to age at which life expectancy was calculated.

The absolute difference in life expectancy between the most and least deprived deciles increased over time for all age groups (Figure 1). In men, the gap increased primarily between 1989-1992 and 1995-1998, particularly at younger ages. Much of the increase between 1995-1998 and 2001-2004 was for life expectancy at higher ages, with little or no increase occurring at very young ages. In women, the increase has been steady throughout the three study periods, with some signs of plateau in 2001-2004 for life expectancy at younger ages.

Table 3 shows overall life expectancy at all ages for men and women, with the corresponding slope index of inequality. In general, absolute inequalities were greater among men than women, although this pattern disappeared at advanced ages, at which point differences between men and women were similar. Even though the slope index of inequality was smaller at advanced ages, absolute differences in life expectancy between most and least deprived areas were progressively important with advancing age relative to overall life expectancy.

**Table 3:** Overall life expectancy (LE) and Slope Index of Inequality (SII) at all ages, Québec, 1989-2004*

Age	Males					
	1989-1992		1995-1998		2001-2004	
	
	Overall LE	SII (%)	Overall LE	SII (%)	Overall LE	SII (%)
0	73.5	3.1 (4.2)	74.7	3.7 (5.0)	76.9	3.7 (4.8)
1	73.0	3.1 (4.2)	74.1	3.6 (4.9)	76.2	3.7 (4.9)
5	69.1	3.1 (4.5)	70.2	3.5 (5.0)	72.3	3.6 (5.0)
10	64.2	3.0 (4.7)	65.3	3.4 (5.2)	67.3	3.6 (5.3)
15	59.3	3.0 (5.1)	60.3	3.4 (5.6)	62.4	3.5 (5.6)
20	54.5	2.8 (5.1)	55.6	3.2 (5.8)	57.6	3.4 (5.9)
25	49.8	2.5 (5.0)	50.9	2.9 5.7)	52.8	3.1 (5.9)
30	45.2	2.4 (5.3)	46.2	2.7 (5.8)	48.0	3.0 (6.3)
35	40.5	2.2 (5.4)	41.4	2.6 (6.3)	43.3	2.9 (6.7)
40	35.8	2.1 (5.9)	36.7	2.4 (6.5)	38.5	2.7 (7.0)
45	31.2	2.0 (6.4)	32.1	2.2 (6.9)	33.8	2.5 (7.4)
50	26.7	1.8 (6.7)	27.6	2.0 (7.2)	29.3	2.3 (7.8)
55	22.5	1.5 (6.7)	23.3	1.7 (7.3)	24.9	2.0 (8.0)
60	18.6	1.2 (6.5)	19.2	1.4 (7.3)	20.7	1.7 (8.2)
65	15.1	1.0 (6.6)	15.5	1.1 (7.1)	16.9	1.4 (8.3)
70	11.9	0.7 (5.9)	12.2	0.8 (6.6)	13.4	1.0 (7.5)
75	9.3	0.5 (5.4)	9.4	0.7 (7.4)	10.3	0.8 (7.8)
80	7.1	0.3 (4.2)	7.0	0.4 (5.7)	7.7	0.8 (10.4)
85	5.5	0.2 (3.6)	5.2	0.4 (7.7)	5.6	0.7 (12.5)
90	4.3	0.5 (11.6)	3.9	0.5 (12.8)	4.2	1.0 (23.8)

**Age**	**Females**					
	**1989-1992**		**1995-1998**		**2001-2004**	
	
	**Overall LE**	**SII (%)**	**Overall LE**	**SII (%)**	**Overall LE**	**SII (%)**
0	80.7	1.8 (2.2)	81.0	1.9 (2.3)	82.2	2.0 (2.4)
1	80.2	1.7 (2.1)	80.4	1.7 2.1)	81.5	2.0 (2.5)
5	76.3	1.6 (2.1)	76.5	1.7 (2.2)	77.6	2.0 (2.6)
10	71.3	1.6 (2.2)	71.6	1.6 (2.2)	72.6	2.0 (2.8)
15	66.4	1.6 (2.4)	66.6	1.6 (2.4)	67.7	1.9 (2.8)
20	61.5	1.6 (2.6)	61.7	1.6 (2.6)	62.7	1.9 (3.0)
25	56.6	1.5 (2.7)	56.8	1.5 (2.6)	57.8	1.8 (3.1)
30	51.7	1.5 (2.9)	51.9	1.4 (2.7)	52.9	1.8 (3.4)
35	46.9	1.5 (3.2)	47.1	1.4 (3.0)	48.0	1.7 (3.5)
40	42.0	1.4 (3.3)	42.2	1.3 (3.1)	43.2	1.6 (3.7)
45	37.3	1.3 (3.5)	37.5	1.2 (3.2)	38.4	1.6 (4.2)
50	32.7	1.2 (3.7)	32.8	1.0 (3.0)	33.8	1.4 (4.1)
55	28.2	1.1 (3.9)	28.3	0.9 (3.2)	29.2	1.3 (4.5)
60	23.9	0.9 (3.8)	24.0	0.8 (3.3)	24.8	1.0 (4.0)
65	19.8	0.8 (4.0)	19.8	0.7 (3.5)	20.6	0.9 (4.4)
70	16.0	0.7 (4.4)	15.9	0.6 (3.8)	16.6	0.7 (4.2)
75	12.5	0.5 (4.0)	12.4	0.6 (4.8)	13.0	0.6 (4.6)
80	9.5	0.5 (5.3)	9.3	0.5 (5.4)	9.7	0.6 (6.2)
85	7.0	0.5 (7.1)	6.7	0.6 (9.0)	6.9	0.7 (10.1)
90	5.3	0.5 (9.4)	4.8	0.7 (14.6)	4.9	0.6 (12.2)

* The percentages were computed by dividing the overall life expectancy with the slope index of inequality multiplied by 100, to reflect the relative importance of inequalities with respect to overall life expectancy.

## Discussion

This study demonstrates that differentials in life expectancy across the lifespan between the most and least deprived areas of Québec have risen over recent decades. These findings support those of other studies examining life expectancy disparities using area-based measures of socio-economic status [1,6,7,11,14]. We systematically evaluated female-male differences and patterns in life expectancy at all ages, this not done in previous studies. Among men, life expectancy differentials between the most and least deprived areas appear to have stabilized over time for younger ages. Among women, differentials seem to be catching up to those of men. Thus, the notion that disparities in life expectancy are greater for men relative to women may not be true for life expectancy at advanced ages in Québec. Our results indicate that, despite gains in life expectancy among men relative to women, differentials between men and women were greater for most deprived relative to least deprived areas in all periods for life expectancy at birth, and in the most recent period for life expectancy at 65 years.

One US study examining area-based trends in life expectancy also reported rising life expectancy differentials between least and most deprived counties from 1980 to 2000, these being greater for men than for women, with better-off areas experiencing the largest absolute gains in life expectancy [11]. Our results indicate similar patterns in Québec. Whether disparities between men and women increased disproportionately in more deprived deciles was not addressed in the US study. The authors did, however, comment on small yet increasing differentials between most and least deprived deciles for life expectancy at advanced ages in both sexes. We observed similar trends in Québec. The US study and our study are not, however, strictly comparable as the study spans differed, other cultural and social differences are present, and Québec CLSCs represent areas of health service delivery while US counties represent areas of local governance.

Studies in New Zealand and the UK suggest either stable [8,12] or increasing [6,7,14] area-based disparities in life expectancy over time (more for men than women). However, these studies primarily examined life expectancy at birth, and did not evaluate age-specific differentials, which could mask emerging inequalities at higher ages [19].

A national analysis of Canadian data observed a decrease in absolute differentials in life expectancy between low and high income neighbourhoods from 1971 to 1996 (although differentials were relatively stable in the 1990s) [15], which contrasts with the results we observed for Québec. The study, however, used census tracts as the unit of analysis, was restricted to urban areas, and excluded institutionalized residents [15]. Other factors such as use of census tracts that changed borders or socio-economic category over time, or for which the attribution of socio-economic category done separately for urban areas which were then pooled for analyses, could have contributed. Alternatively, it is possible that relationships are different in urban areas of Canada, or that residual confounding is present in our study even though we adjusted for rurality. However, other recent studies of Canadian provinces have documented increases in deprivation-related area-based premature mortality, including urban areas of Québec with the exception of Montréal [4,5]. Relationships at the provincial level may also be different than those at the national level.

Our study was not designed to test the mechanisms underpinning the rise in life expectancy differentials related to area deprivation. We nonetheless verified that differential population growth, which has been highlighted as a possible explanation [36], had no impact on summary measures of inequality. Income inequality has also been proposed as an explanation [6,11]. However, a recent ecologic study showed no association between income inequality and mortality in Canada [37]. Although these associations have yet to be confirmed in a multi-level study, it is nevertheless interesting that income inequality increased over the course of our study [38]. Last, selective migration of healthy (or unhealthy) people into areas with greater or lesser material deprivation remains a plausible explanation [6,7].

Causes of death [5] such as accidents, suicide and violence [7] that vary differentially by gender between more and less deprived areas may explain the generally greater differences observed in this study among men, particularly at younger ages. Health-related behaviours might also be less favourable in men than in women, especially at younger ages [7]. The growing use of tobacco by women might explain why disparities among women continued to increase rather than stabilise. Despite Québec's universal health care insurance, changes in health service accessibility and/or availability might also contribute to rising differentials [28]. The widening gaps observed at advanced ages (when medical care is most often sought) [39], suggests the health care system may be better serving less deprived areas of Québec.

This study was subject to limitations. Areas were classified according deprivation data from the central study period, which might misestimate area-based inequality in other study periods [36]. However, any area misclassification was likely minor, and unlikely to change the relative ranking of areas in the first and last study periods [40]. We could not exclude institutionalized deaths and residents. Life expectancy for advanced ages should be interpreted with caution because of small sample sizes which might result in less stable estimates. Although we accounted for rurality and Aboriginal reserves, we did not evaluate other area characteristics that might explain our findings such as immigration or ethnicity; however, immigration is highly correlated with rurality which is already accounted for in the analyses, and previous CLSC-based research also suggests immigration is not a confounder of the relationship between area socio-economic status and mortality or adverse birth outcomes [31,32]. We used a relatively large definition of areas (CLSCs), and it is possible that the use of smaller areas may have yielded different results. Last, our results can only be used to understand population-level trends, not for individual-level inference.

## Conclusions

In Québec, differentials in life expectancy across the life span between the most and least deprived areas are present and rising in both sexes, and are comparable to differentials present in the US. Disparities are relatively large for women of advanced ages. Previous research indicating that life expectancy differentials between most and least deprived areas are greater in men may be due to a focus on younger age groups. Additional studies are necessary supplemented with alternate methodologies to determine the reasons for widening differentials in order to better influence health policy. Because disparities in life expectancy are increasing despite universal health care, a focus on health care alone is unlikely succeed in reducing disparities in health.

## Abbreviations

CLSC: (Local community services centre).

## Competing interests

The authors declare that they have no competing interests.

## Authors' contributions

NA and CA conceived and designed the study. CA reviewed the literature. NA guided and CA/GZ performed the data analyses. NA and CA interpreted the results and wrote the manuscript. MD helped interpret the results. GZ and MD revised the manuscript for important intellectual content. All authors approved the final version of the manuscript.

## Pre-publication history

The pre-publication history for this paper can be accessed here:

http://www.biomedcentral.com/1471-2458/10/161/prepub
